# A meta-analysis of the efficacy of HAART on HIV transmission and its impact on sexual risk behaviours among men who have sex with men

**DOI:** 10.1038/s41598-019-56530-8

**Published:** 2020-01-23

**Authors:** Ying Jiang, Shu Su, Yan Borné

**Affiliations:** 1 Department of Clinical Sciences in Malmö, Faculty of Medicine, Lund University, Lund, Sweden; 2 School of public health and preventive medicine, Monash University, Melbourne, Australia

**Keywords:** HIV infections, Medical research

## Abstract

Evidence showed preventive impacts of the highly active antiretroviral therapy (HAART) on the Human Immunodeficiency Virus (HIV) transmission amomg heterosexual population, however, that is of deficit among men who have sex with men (MSM). The aim was to systematically examine the efficacy of HAART on HIV transmission and the association between the HAART initiation and unprotected anal intercourse (UAI) in MSM population. Three electronic databases were fully searched for articles published in peer-reviewed journals between 1996 and 2017. Of 1616 identified articles, fifteen articles were eligible for meta-analyses. The summary incidence rate (IR) of HIV was 6.63/100 person-year (95%CI 2.06–11.20/100 person-year)(p = 0.004). The pooled per-contact rate (PCR) of HIV was 0.42% (95% CI 0.21–0.63%)(p < 0.05). The HAART initiation (vs non-HAART) was not associated with engaging in UAI, with odds ratio (OR) 1.09 (95% CI 0.90–1.34)(p > 0.05). In the stratified analysis, participants with no less than 6 months recall period was slightly more likely to engage in UAI (OR 1.32; 95% CI 1.01–1.74)(p < 0.05). It indicated that HAART has potential efficacy on reducing infectivity of HIV positive individuals in anal intercourses. The relationship between the HAART initiation and UAI was not significant and may be influenced by some social-demographic factors. Consistent condom use and education on safe sex among MSM are crucial.

## Introduction

Globally, over 78 million people were infected with HIV. The prevalence of people living with HIV was 36.7 million^1^. Among men who have sex with men (MSM), the average percentage of HIV prevalence ranged between 6% (the Asia and Pacific region) and 15% (the western and central Africa region)^2^. In young (under 25) MSM, around 4.2% of the population was infected with HIV^2^. Although there is a trend of reduction of HIV incidence in the general population in most countries, it showed an upward trend among MSM^2,3^. Two Australian studies predicted that, for homosexual males, the mean number of homosexual partners was 11 in the past 1 year^4^, compared with heterosexual men, which was about 1.5^5^. Theoretically, with high possibility of partner change rate, the MSM is regarded as the core group in the transmission of sexual transmitted diseases (STIs) including HIV^6^. Also, the MSM-population is supposed to be the risk group in the pattern of HIV transmission. A UNAIDS report estimated that the risk of HIV acquisition in MSM was 19 times higher than that in the heterosexual population^2^. Compared with different patterns of sexual intercourses, the risk of HIV transmission was the highest via the UAI^7^.

The multiple combination of antiretroviral drugs (ARVs), highly active antiretroviral therapy (HAART), was proved to successfully suppress the HIV virus in blood^8^. A landmark randomized clinic trail (RCT) HPTN 052 demonstrated that the HAART reduced individuals’ risk of HIV-1 transmission via sex with condoms by 96% between heterosexual sero-discordant partners^9^. Similar results were found in an observational cohort study in Africa^10^. However, both studies only detected the effect of HAART on the vaginal transmission, whether the HAART reducing the risk of HIV acquisition via penis-anal intercourses is unknown. Some researchers believed that the rectal tissue is more susceptible to HIV RNA virus than vaginal tissue, which would increase individuals’ risk of HIV acquisition^11^.

In 2012, WHO published a guideline regarding HAART as a secondary preventive strategy called the treatment as prevention (TasP) based on previous empirical evidences^12^. However, the preventive effect of HAART on MSM population is uncertain and may be influenced by many factors. For instance, some researchers pointed that the unstable and weak relationship between MSM may avert the preventive effect of the HAART^7^. In addition, the viral load remained at high level in rectal tissues, even though it has been suppressed at an undetectable level in plasma^13,14^. Hence, behavioural factors like condomless anal intercourses may induce negative impact on the TasP^15^. In contrast, a prospective cohort study illustrated none HIV negative participants were linked-infected by their HIV positive partners via unsafe sex^16^.

It is imperative to fill the gap with specific evidences of the efficacy of TasP on HIV transmission in MSM. Detecting the association between UAI and HAART and potential influential factors for UAI would help researchers and policy-makers tailored their public health projects for this sub-group population. In this meta-analysis, the term *HIV* is represented the HIV-1 type infection.

The aim of this systematic review and meta-analysis was twofold: 1) to examine the efficacy of HAART on the risk of HIV transmission among MSM, 2) to examine the likelihood of engaging in sexual risk behaviours which measured as UAI among MSM while HAART initiation.

## Results

### Characteristics of studies

Eighteen studies published between 2001 and 2006 were initially involved in the meta-analysis. Excluding 3 qualitative researches, 7 cross-sectional studies and 8 cohort studies were finally rolled in further analysis, contributing to an overall study population of 26040. Data were collected at clinical sites or gay communities via self-administrated questionnaires and medical records. Over half of the studies (8/15) adopted the 6-month recall period. Three articles^17–19^ from the same prospective national cohort were also included due to different study periods and samples (Table 1). The median of sample size (n = 14) was 714.5 (IQR 1410), ranging from 155 to 12573. Researches mainly conducted in developed countries (*i.e*. UK, Australia and USA, n = 12). The majority of the participants (91.83%) were HIV positive status. The median age of MSM (n = 12) ranged from 28 to 45, with mean age 35.7 ± 4.44 y. Ten studies reported ethnicities of participants, mainly consisting of white (80%, n = 16150), the others were Asians, African Americans and Latino/Hispanic. Nearly half of the participants held the degree of college or above (41%, n = 10932). However, few studies reported the income level (n = 4), alcohol intake (n = 4) and substance use (n = 5) and the number of sexual partners ((n = 4) (Table 2).

**Table 1:** Basic information of eighteen study designs.

Author and Years	Locations	study design	study setting	data collection methods	Study period	Sample size for analysis	Recall period (within past months)	Response rate (%)	Length of follow-up
Brennan. DJ. 2010	USA	cross sectional study	community	self-reported questionnaires	unknown	346	12	Not report	—
Cowan. SA. 2012*	Demark	cohort study	gay community	self-reported questionnaires	Jan. 1995–Jan. 2010		—	—	—
Cox.J. 2004	Canada	cross sectional study	5 ambulatory HIV clinics	self-reported questionnaires	Oct. 2002–Feb.2003	346	6	50	—
Cunha. CB. 2014	Brazil	cross sectional study	1 clinic	self-reported questionnaires, medical records	Aug. 2010–Jun. 2012	155	3	93.2	—
Dukers. NH. 2001	Netherland	cohort study	clinics	self-reported questionnaires, medical records	Jan. 1992–Jan. 2000	365	6	—	Unclear, probably 1 year
Dukers. NH. 2002	Netherland	cross sectional study	STD clinics	self-reported questionnaires, medical records	1999–2001	3090	6	Not report	—
Fisher. M. 2010*	UK	cohort study	1 clinic	self-reported questionnaires, medical records	2000–2006	—		—	—
Gorbach. PM. 2011	USA	cohort study	clinics	self-reported questionnaires, medical records	2002–2006	187	3	—	12 months
Jansen. I. AV. 2011	Netherland	cohort study	Public Health Service of Amsterdam	self-reported questionnaires, medical records	Oct. 1984–Dec. 2009	1642	6	—	11223 person-year
Jin. F. 2010	Australia	cohort study	gay community	self-reported questionnaires, medical records	Jul. 2001–Jun. 2007	1136	6	—	5160 person-year
Magidson. JF. 2015	Latin America (*i.e*. Argentina, Brazil, Chile, Columbia, Mexico, Peru, Venezuela)	cross sectional study	community	self-reported questionnaires	Oct. 2012- Nov. 2012	2350	3	79.8	—
Mori. SF. 2005	USA	cross sectional study	community agent and clinics	self-reported questionnaires	unclear	1870	3	Not report	—
Porco. TC. 2004*	USA	cohort study	community	self-reported questionnaires	1994–1999	—	—	—	—
Rodger. AJ. 2016	European countries (*i.e*. UK)	cohort study	75 clinics	self-reported questionnaires, medical records	Sep. 2010–may. 2014	680	6	—	1238 couple-year
Safren. SA. 2016	Thailand and Brazil	cohort study	clinics	self-reported questionnaires	Mar. 2011–May. 2013	749	2	—	15 months
Scott. HM. 2014	UAS	cohort study	not known	self-reported questionnaires	1992–1999	12573	6	—	18 months
Stephenson. JM. 2003	UK	cross sectional study	an outpatient clinic	self-reported questionnaires	Jul. 1999–Aug. 2000	405	12	97.9	—
Stolte. IG. 2004	Netherland	cohort study	Multiple Health Service	self-reported questionnaires	Sep. 1999–May. 2002	146	6	—	21.6 months

^*^Studies were not included in meta-analysis.

**Table 2:** Characteristics of studies tested the efficacy of HAART on HIV transmission.

Author and Years	ethnicity (%)	age (mean/median) Y(range/IQR)	sero-status of participants	education (college or above, n)	heavy alcohol user*(n)	income	substance use	number of sexual partners during recall period
Dukers. NH. 2002	77.1 Dutch	34(28–40)	positive and negative	not report	not report	not report	not report	not report
Jin. F. 2010	unclear	35(18–75)	negative	not report	not report	not report	unknown	not report
Rodger. AJ. 2016	89.1 white, 0.9 Afrian American, 8.8 Asian and others	HIV positive 41.7(35.5–46.8); HIV negative 40.1(31.9–46.5)	sero-discordant couples	339/680	not report	not report	not report	not report
Safren. SA. 2016	not report	30–49	positive	not report	Unclear (OR 1.04)	not report	not report	not report
Scott. HM. 2014	79.4 white, 5.3 African American, 10,9 Latino, 4.3 Asian and others	unclear	negative	7884/12573	2219/12573	not report	2504	mainly over 5

### HAART and HIV IR

**Figure 1:**
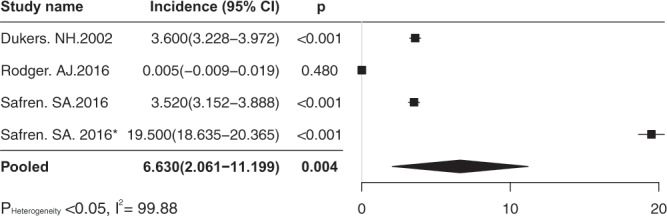
The forest plot of pooled estimate of IR, 100 person-year, in the era of HAART.

Four effect sizes contributing from three independent studies tested the IR of HIV in the era of HAART. The overall estimate of IR was 6.63/100 person-year (95% CI 2.06–11.20/100 person-year), p = 0.004 (Fig. 1). The heterogeneity test showed wide heterogeneous (p < 0.05; I^2^ = 99.88), thus the random-effects model was used. Egger’s regression intercept test showed no publication bias (p > 0.05). Sensitivity analysis showed no single effect size influenced the overall result.

### HAART and PCR

**Figure 2:**
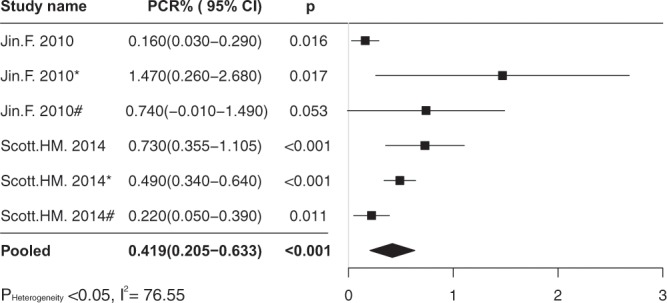
The forest plot of pooled estimate of per-contact rate (%) in the era of HAART.

Two studies contributing six independent effect sizes examined the PCR of HIV transmission in the era of HAART. The pooled estimate of PCR was 0.42% (95% CI 0.21–0.63%), p < 0.001 (Fig. 2). The heterogeneity test showed substantial heterogeneous across effect sizes (p < 0.05; I^2^ = 76.55). The random-effects model was used. Egger’s regression intercept test showed no publication bias (p > 0.05). Sensitivity analysis showed the summary PCR did not influenced by removing each independent effect size.

### HAART and UAI

**Figure 3:**
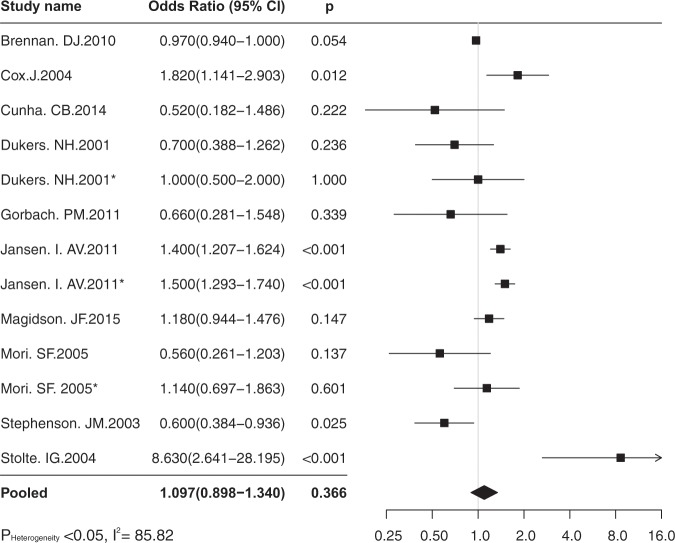
The forest plot of pooled estimates of association between HAART and UAI.

Ten studies contributing thirteen independent effect size tested the association between HAART (vs not HAART) and risk sexual behaviours. The percentage of HIV positive participants on HAART ranged from 23% to 100%. Only 2 studies^17,20^ reported the average length of HAART initiation (70 months and 10.8 months). Four studies presented the average duration of HIV infection, varying from 5.5 (IQR 0.2–16.4) to 12 (IQR 7–6) years^20–23^ (Table 3). The pooled estimate of OR was 1.09 (95% CI 0.90–1.34, P = 0.366), p > 0.05 (Fig. 3). The heterogeneity test showed wide heterogeneous across studies (p < 0.05, I^2^ = 85.82), thus, the random-effects model was used. The overall effect size was not influenced by removing single effect sizes when running the sensitivity analysis. There was no evidence of publication bias investigated by the Egger’s regression intercept (p > 0.05).

**Table 3:** Findings of independent studies.

Author and year	Measurement of effect size	Findings
Brennan. DJ. 2010	OR	Examination conducted between HAART belief of individuals on HAART and UAI. Scale contains 3 items, of which condom motivaion examined the association about perosonal attitudes towards condom use while on HAART with UAI. OR: 0.97 (95% CI 0.94–1.00)
Cowan. SA. 2012	incidence (absolute number)	Yearly incidence of HIV diagnosed MSM in the Danish Cohort Study: median number 93/per year (range 71–137).
Cox.J. 2004	Adjusted OR	84% participants were on ART, 79% had at least one sexual partners, 194 anal sex, of which 93 with HIV positive, 72/93 UAI, 101 with HIV negative or unkown partners, 45/101 UAI. OR (UAI vs PI) 0.52 (95%CI 0.33–0.83), OR (UAI vs NRRIT) 1.95 (95%CI 1.00–3.80). AOR (UAI vs ART) 1.82 (95%CI 1,14–2.90)
Cunha. CB. 2014	Adjusted OR	63 UAI vs 92 non-UAI. 126 were on ART, of which 80 were non-UAI, 46 were on UAI. (HAART was remained as a confounder variable in multivariate model). AOR(ART vs UAI) 0.52 (95% CI 0.18–1.47)
Dukers. NH. 2001	OR	OR (ART vs UAI with Steady partners) 0.7 (0.4–1.3), OR (ART vs UAI with casual partners) 1.0 (0.5–1.9)
Dukers. NH. 2002	Annual HIV incidence (new infection/HIV negative and recent infection)	Overall HIV prevalence 14.7(454/3090). Incidence: 3.0 infections/100 person-year (95%CI 1.8–4.6)
Fisher. M. 2010	RR	RR (HAART vs HIV transmission risk) 0.14 (95% CI 0.07–0.27)
Gorbach. PM. 2011	OR	OR(HAART vs UAI): 0.66 (95% CI 0.28–1.54)
Jansen. I. AV. 2011	OR, IRR(incidence risk ratio)	217 of 1642 MSM seroconvetion, counted as yearly incidence/100 person-year, counted as yearly rate of UAI. OR (UAI vs HAART): 1.4 (95% CI 1.16–1.56) 1996–2003, 1.5 (95% CI 1.33–1.79) 2003–2009, compared with 1992–1996(pre-ART). Proportion UAI with steady patner was 60%, with casual partners 26% in 2009. In 180 sero-converted, 134 was allocated to a casual partners and 46 was allocated to the steady partners.
Jin. F. 2010	PCR, OR	46 sero-conversion. Total episode of UAI 228056. counted UAI with HIV positive, negative and unknown partners with three types of sex. PCR for insertive UAI with circumcised 0.11% (95% CI 0.02–0.24), and 0.62% (95% CI 0.07–1.68) without circumcised. PCR for receptive UAI with ejaculation inside OR 1.43 (95% CI 0.48–2.85), withdraw OR 0.65 (95% CI 0.15–1.53). Regardless of circumcision, PCR for insertive UAI was 0.16% (95% CI 0.05–0.31), receptive UAI with ejaculation inside was 1.47% (95% CI 0.51–2.93) and withdraw was 0.74% (95% CI 0.18–1.68)
Magidson. JF. 2015	AOR(adjusted for sociodemographic characteristics	Among 2350 HIV positive MSM, 684 not on ART, 1666 on ART. 949 not took UAI, 848 took UAI. AOR (ART vs UAI with sero-different partners) 1.18, 95% CI 0.94–1.47). OR (UAI vs 100% adherence of ART) 1.55
Mori. SF. 2005	OR	OR (ART vs steady partner UAI) 0.56 (95% CI 0.26–1.20), OR (ART vs casual partner UAI) 1.14 (95% CI 0.70–1.87)
Porco. TC. 2004	transmission probability per partnership	534 uninfected participants at baseline. Transmission probability per-partnership was 0.0276 on pre-HAART, and 0.011 on post-HAART was 0.048.
Rodger. AJ. 2016	Rate of sero-conversion,	10 MSM couples, 1 heterosexual couples. But non-linked sero-conversion happened (0%)
Safren. SA. 2016	probability of transmitting HIV	Estimated HIV transmission per 100 persons in Thailand was 3.52%, in Brazil was 1.95%.
Scott. HM. 2014	per-contact risk	In the pre-HAART era, 52/1813 seroconversions. In the early HAART era, 584/42395 seroconversions. With HIV positive partners, receptive UAI 0.60 (95% CI 0.34–1.09). Estimated PCR of receptive UAI with seropositive partners in pre-HAART: 0.6% (95%CI 0.34–1.09%), early HAART 0.73% (95% CI 0.45–0.98%)
Stephenson. JM. 2003	OR	113 were not on HAART, 292 were on ART. UAI (on ART) 101/285, UAI(not on ART) 51/107; insertive UAI (on ART) 76/285, insertive UAI (not on ART) 39/107. OR (UAI vs HAART in the past 12 months), 0.60 (0,39–0.95).
Stolte. IG. 2004	adjusted OR	(reference group: non-UAI with casual partners) Adjusted OR(ART-ralated belief vs UAI with casual partner) 8.63 (95% CI 2.64–28.18)

By previous literature review, we stratified data into sub-analyses (Table 4). On HAART group (vs non-HAART) with the recall period over 6 months had more slightly likelihood of engaging in UAI (OR 1.32; 95% CI 1.01–1.74, p < 0.047). While the other factors (*i.e*. study setting, Data collection method) remained none association between two variables (HAART and UAI).

**Table 4:** Stratified analysis for UAI and HAART.

Variables	Number of effect size (n = 13)	OR(95% CI)	p value	Heterogeneity	
				p value	I-square
**Study setting**					
Clinics	11	1.09(0.84–1.42)	0.5	0.00	76.68
Non-clinics	2	1.04(0.86–1.24)	0.71	0.09	65.47
**Data collection method**					
Self-reported	7	1.11(0.84–1.47)	0.47	0.00	79.85
Self-reported & medical records	6	1.16(0.91–1.48)	0.22	0.02	62.596
**Recall period**					
Less than 6 months	6	0.82(0.58–1.15)	0.244	0.03	58.19
More than 6 months(include 6 months)	7	1.32(1.01–1.74)	0.047	0.00	91.729
**Sero-status of participants**					
Positive only	10	0.95(0.79–1.15)	0.61	0.02	55.24
Negative with(out) positive	3	1.60(1.12–2.12)	0.001	0.01	77.96
**Median age**					
Less than 35	4	1.45(1.16–1.82)	0.001	0.00	75.22
More than 35 (include 35)	9	0.90(0.71–1.13)	0.35	0.03	53.39
**Sample size**					
Less than 300	3	1.39(0.28–6.96)	0.69	0.00	86.63
More than 300 (include 300)	10	1.09(0.90–1.33)	0.37	0.00	87.05

## Discussion

### Effects of HAART on HIV infectiousness

Few international studies examined the IR among MSM before HAART therapy adopted. Therefore, comparable groups’ data (non-HAART) in most researches were absent.

In general, the pooled IR (6.63/100 person-year, p < 0.05)) in our meta-analysis was within the range of the incidence rate of HIV (1.2 to 14.4/100 person-year) in MSM population reported by WHO^24^. The overall weighted IR was highly influenced by the number contributed from Brazil^25^. This extreme data indicated that the risk of HIV transmission among MSM may be different within distinct regions. However, the assumption, whether the diversity of IR came from different samples or it was only an extreme case, needs further proof.

A prospective cohort study among four effect sizes demonstrated that the HAART had a preventive effect on HIV transmission via condomless anal intercourse at the individual-level^16^. It moderated the infectivity of HIV positive individuals^17^. However, at the population level, studies calculated the HIV IR in the era of pre-HAART and post-HAART seperately showed an increasing trend during 1991 and 2001 in Netherland^26^. This contradicted phenomenon revealed that there might be some environmental factors around MSM influence the preventive impact of HAART, even averted the preventive efficacy at this population.

The PCR refers to the probability of one person be infected by their sexual partners while exposed at a certain sexual pattern, measured as infectivity (β) of HIV^7^. Porco and colleagues (2004) calculated 60% decrease of the PCR of per-partnership in the post-HAART era compared with that in the pre-HAART era^27^. An across-country prospective cohort study (*Opposites Attract study*) investigated that the HAART had a positive impact on diminishing the infectivity of HIV viral hosts by suppressing the viral load in the plasma under 200 copies/ml (0 linked-infection, unpublished data), which is similar with that found by Rodger. AJ *et al*.^16^. Those optimistic findings were only shown between steady sexual relationships. However, evidences displayed that homosexual males were more prone to be involved in polygamous and vulnerable relationships^4^, which may contribute part of reasons for the uncertain efficacy of HAART on MSM. In addition, this research showed that the overall β of UAI is not zero even the HAART initiated. Thus, keep encouraging condom use among MSM is indispensable to prevent the HIV transmission. Researches have been proved that the preventive impact of condom use on HIV/STI transmission was effective if a person practices sexual activities with condoms all the time^28^. Consistently expanding HAART on MSM could be one approach to control the epidemic of HIV, which potentially decreases the average infectivity of the total population. Monitoring and guaranteeing the adherence of HAART intake should be a supplementary approach to maximise the impact of HAART.

### Association between HAART and UAI

The pooled effect size of the third meta-analysis showed the HAART would not influence the people’s choice on condom use during sexual activities. However, in the stratified analysis, participants had sexual experience no less than previous 6 months were more likely to engage in UAI (p = 0.047) compared with that within past 3 months, which implies that the frequency of sexual intercourses may influence the association between UAI and HAART. On the other hand, there may be a recall bias, since participants with longer recall period had higher chance to be vague on the memory of past sexual experiences. The reduction of self-reported reliability of sexual behaviours was also reported in the study of Napper (2009), which mentioned this reduction could be detected if the recall period beyond 6 months^29^. However, Napper pointed out that this finding needs further proof on anal intercourses^29^.

Confounders in the third meta-analysis were probably multiple and probably involve both socio-demographic and individual sides. The age, income and educational level, alcohol and drug abuse have been found to be significantly associated with engaging in UAI, indicating that people are at both young and old age, with lower education, successive alcohol drinking and substance (*i.e*. “popper”) use before or during sex were more likely to engage in UAI^18,20–22,30,31^. The high income was found to be the risk factor for engaging UAI^21^. However, the lower income group held optimistic beliefs on the preventive impact of HAART^20^. Some researchers believed that people held positive beliefs on the HAART was more likely to engaging in UAI^17,20^. This psychological construction also found in the research of Huebner. DM and Gerend. MA. (2001), pointing out that the HIV infected MSM who believed HAART has the preventive capability were more prone to take UAI, especially with casual sexual partners^32^. Therefore, consistent providing knowledge about safe sex, such as the limitation of TasP strategies and the importance of condom use in anal sex, is essential in the post-HAART era. However, the “condom fatigue”, referring to people get tired of health education on condom use and lag to change sexual risk behaviours, was mentioned in both Cox. J. (2004) and Brennan. DJ. (2010) researches, which may decrease the efficacy of safe sex education in the long-term run^20,21^.

## Conclusion

Even though the information of HAART on MSM is limited and HAART preventive efficacy on HIV transmission among MSM was hardly draw a robust conclusion at this stage, this meta-analysis was the first aggregated quantitative research focused on the HIV transmission among MSM and provided specific information of this public health issue. Individual epidemiological findings have illustrated an optimistic opportunity for TasP to control the epidemic of HIV in community. However, since related information was scarce, further researches could emphases on its efficacy on population-level and detect potential influential factors. Also, data were mainly contributed from open societies like the USA and Europe, researchers and public health policy-makers from other regions should interpret those findings carefully in local contexts. In our meta-analysis, we reviewed social and individual factors that may confound the relationship between HAART and UAI. However, the impact of characteristics of HAART was not mentioned, including the adverse effect and drug resistance. Therefore, we encourage future studies could be designed more comprehensively to explore the relationship between HAART and UAI in MSM.

## Limitations

There are some limitations in this meta-analysis. First, this meta-analysis did not include unpublished articles due to limited accessible literature resources. Also, we excluded non-full articles because we did not access original data. Second, there are wide heterogeneities. We did not perform a stratified analysis for the meta-analysis of the HAART efficacy since the total number of effect sizes was too few to conduct the sub-group analysis. However, we conducted a stratified analysis for the third meta-analysis to examine the influential factors. Third, participants included in the meta-analysis were skewed on western countries and open societies. In eastern countries, cultures and social structures would be different. For instance, in some countries in Africa and Asia, admitting self-identities as gay are still illegal^2^. Hence, findings of this meta-analysis need carefully and cautiously to generalize into different contexts. In addition, due to few HIV data of MSM collected in Asian and African countries, we encourage further researches to turn eyes on investigating reasons behind this phenomenon, help policy-makers in those countries formulate public health policies on HIV interventions and devote to changing this social issue. Fourth, we reviewed the social and individual factors that may confound the relationship between HAART and UAI, however, the impact of characteristics of HAART was not mentioned, including the adverse effect and drug resistance. Fifth, there are a few biases in the meta-analysis. Data were collected from self-reports, which would introduce a recall bias. Participants recruited from clinics may introduce the selection bias, since people who attended to clinics may be more care about their health and this self-awareness may overestimate the efficacy of HAART on the HIV incidence and sexual behaviours in the overall MSM population.

## Materials and Methods

### Screening and inclusion criteria

The following criteria were carried out for literature research:

1. Participants were over 18 years old (including 18) or defined as adults according to National Laws.
2. Participants were homosexual males, self-identified as gay or engaging in male-to-male sexual behaviours regardless of original sexual orientation.
3. HAART were exposed on HIV positive rather than negative participants.
4. Studies performed to examine 1) the risk of HIV transmission, and/or 2) any types of UAI (*i.e*. receptive or insertive).
5. Studies published as journal articles in English with peer reviewed.
6. Full text articles are available online.
7. Articles published between January 1996 to February 2017.

### Search strategies and literature research

Electronic database PubMed, ScienceDirect and Google Scholar were fully searched. Terms‘HIV’, ‘antiretroviral’, ‘transmission’, ‘men who have sex with men’, ‘homosexual’, ‘behaviours’, ‘treatment as prevention’, ‘test and treat’, ‘anal sex’ were crossly combined and searched as either keywords in titles and abstracts or Medical Subject Heading (MeSH) terms (*i.e*. ‘HIV’ and ‘behaviour’). The literature research was conducted mainly in the form of two search strings: (1) ‘HIV’, ‘antiretroviral’, ‘transmission’, (‘men who have sex with men’ OR ‘homosexual’); (2) ‘HIV’, ‘antiretroviral’, (‘men who have sex with men’ OR ‘homosexual’), ‘behaviours’. Keywords ‘test and treat’, ‘treatment as prevention’ ‘anal sex’ were performed as supplementary search strategies with the other terms. Keywords ‘pre-exposure’, ‘hepatitis’, ‘HPV’, ‘herpes’, ‘drug resistance’ and ‘prevalence’ were excluded from title and abstract while searching at the database of Google Scholar. The search period was adopted from January 1996 to the present (February 2017).

### Screening

2327 articles were found and 1616 articles were remained for further examination after removing duplications. Titles or abstracts were excluded if relating to:

1. irrelative study purposes: psychological and mental health,virology, health economics and policy, other STIs and AIDS-related diseases, drug resistance, belief or attitudes towards safe sex rather than behavioural changes, characters of participant, HAART scaling-up strategies.
2. irrelative exposures (HAART) or study groups: PrEP or PEP, alternative interventions (*i.e*. behaviours, partner notification, testing and counselling),heterosexual or women participants only.
3. irrelative study design: molecular epidemiology, qualitative researches, grey articles, news and reviews.

As a result, 75 articles were left for eligibility assessment via full text reviewed. The following principles were used to guide the full text article screening of this meta-analysis:

1. Studies performed to examine the efficacy of HAART on HIV transmission and/or the relationship of HAART and the UAI irrespective types of sexual patterns and partners.
2. Studies were focused on the change of actual sexual behaviours rather than the change of attitudes towards sexual behaviours.
3. If participants were made up of mixed subgroups (*i.e*. homosexual, heterosexual and bisexual), the subgroup of homosexual males or male couples would be included in this meta-analysis.
4. If the database overlapped between articles, with similar research purposes, the one contained the widest range of data would be included.
5. Outcomes were measured for HIV incidence or the likelihood of engaging UAI.
6. The ecological studies would not be included, because exposures were multiple and aggregated, potential confounders and the ecological fallacy may contribute great heterogeneity to the pooled analysis^33^.

**Figure 4:**
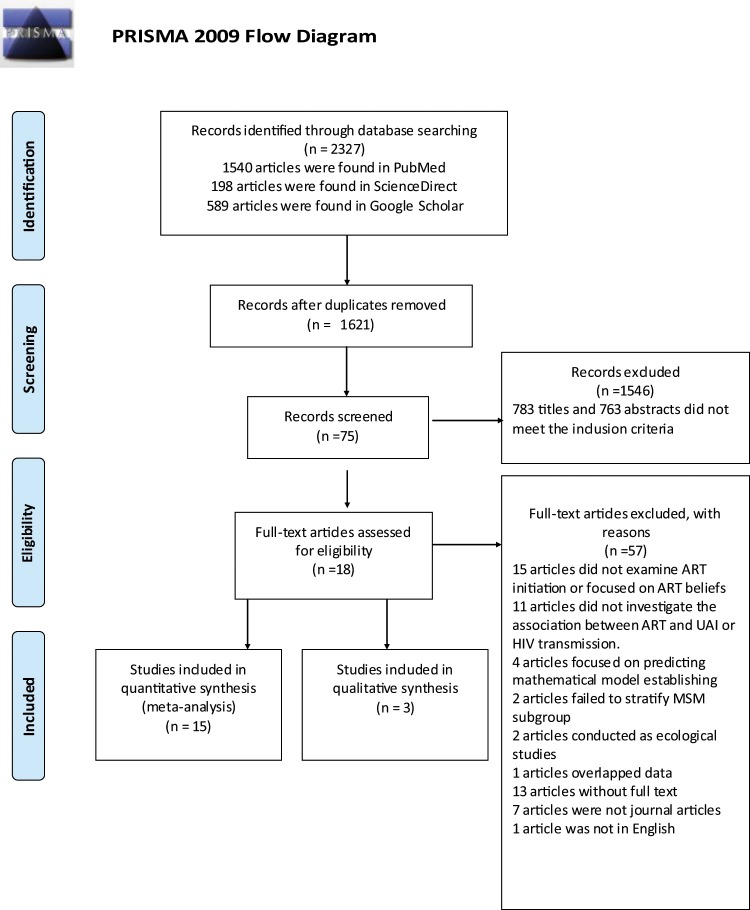
PLASMA flow chat of literature search.

57 articles were excluded via full text screening with following reasons listed in the Fig. 4. Finally, eighteen articles were included in this quantitative analysis. However, data presented in three articles were not eligible for meta-analyses^27,34,35^. Two studies measured by the incidence per year, which was not comparable with other studies^34^. The other study measured the association between HAART and HIV transmission risk by rate ratio (RR) was hardly fitted into the third meta-analysis^35^. Thus, there were fifteen full text articles enrolled in the final data analysis. This systematic review was carried out according to guidelines by PRISMA checklist^36^. The PRISMA flaw chat for the data selection procedure has been followed and presented in Fig. 4.

### Data extraction

Each study was numbered by the first author and published year. A unified form was designed for the data extraction and imported into a Microsoft Excel database, including following categories:

1. Study design characters: authors and years, location, study design, study setting, data collection methods, study period, sample size for analysis, recall period (within past months), response rate, length of follow-up (person-year);
2. Participants’ characters: ethnicity, age, sero-status of participants, education, income, heavy alcohol users, substance use, number of sexual partners during recall period, since when diagnosed, type of data analysis;
3. Findings: measurement of effect size and findings.

If both adjusted and unadjusted effect sizes were reported by independent studies, the unadjusted one would be included for the meta-analysis. Additionally, if the effect size was stratified by regions or types of sexual patterns, the sub-level number would be added into the meta-analysis. The extracted items listed in standard forms (Tables 1, 2, 3 and 5) were tested by three different pilot studies^21,22,37^.

**Table 5:** Characteristics of studies tested the association between HAART and UAI.

Author and Years	ethnicity (%)	age (mean/median) Y(range/IQR)	sero-status of participants	education (college or above, n)	income	alcohol (n)	substance use	median number of sexual partners during recall period	since when HIV diagnosed (median/mean (IQR/range), y)	type of data analysis
Brennan. DJ. 2010	23 white, 4.6 African American, 47.5 Hispanic, 24.6 others	43(38–48)	positive	217	not report	109 (never), 129 (less 3 times/months), 104 (1–2 times a week or per day)	143 never, 203 at leat once (in the past 3 months)	not report	12(7–16)	multi-variate analysis
Cox.J. 2004	Unclear	45(24–73)	positive	111	35% (<$20,000), 33%($20,000–39.,999), 32% (>=$40 000)	not report	not report	36% had 6 or more partners	10(0–19)	multi-variate analysis
Cunha. CB. 2014	52.9 White	38(32–45)	positive	128	not report	36.8% high use in the past 3 months	57 use before sex in the past 3 months	commercial sex	6(2.8–12.4)	multi-variate analysis
Dukers. NH. 2001	88.4 central and European white	39.9(31.4–42.8)	positive	145	not report	not report	not report	not report	not report	multi-variate analysis
Gorbach. PM. 2011	71 white,21 Hispanic	35(19–64)	positive	165	not report	not report	21.1 use at last sex	8.8 in the past 3 months	nuclear	multi-variate analysis
Jansen. I. AV. 2011	81 Dutch	28.8(24.8–35.9)	negative	901	not report	not report	not report	unclear	not report	multi-variate analysis
Magidson. JF. 2015	Unknown	28(23–25)	positive	78.2%	74.4% middle income class	not report	not report	not report	not report	multi-variate analysis
Mori. SF. 2005	38.4 white, 35.3 African American, 17.8 Latino, 8.34 Asian and others	40	positive	1153	1212 unemployment, 658 employment	620 none, 1145 some, 100 daily	331 use, 1538 non-use	not report	not report	multi-variate analysis
Stephenson. JM. 2003	90.3 white	38(21–64)	positive	228	199 full-time, 77 unemployment, 43 medically retired, 43 other	not report	not report	12 in the past 12 months	5.5(0.2–16.4)	multi-variate analysis
Stolte. IG. 2004	93.1 Dutch	30.8(26.3–33.5)	negative	unclear	not report	not report	not report	not report	not report	multi-variate analysis

### Data analysis

Three independent meta-analysis were performed. The pooled risk of HIV transmission was measured separately by the IR (the number of new infections divided by at risk population over specific time) and the PCR (estimation based on Bernoulli model or boost rapping algorithm likelihood function) with 95% CI. The pooled OR with 95% CI was used to estimate the association between HAART and UAI, representing the probability of engaging UAI between the on-HAART group (index group) and the non-HAART group (reference group). The baseline data in cohort studies were included in meta-analysis. If OR is over 1, it represented that the index group is more likely to engage in UAI than the reference group. Otherwise, the index group is less likely to engage in UAI (OR < 1). If OR = 1, it means there is no association between both variables.

A Cochran’s Q test based on Chi-square test and an I^2^ test were used to examine the heterogeneity across independent studies. The fixed-effects model would be used if the data showed low or moderate heterogeneity (p > 0.05, I^2^ < 50%), otherwise, the random-effects model (p < 0.05, I^2^ > = 50%) would be carried out^38^. Publication bias was tested by the Egger’s regression intercept, if p > 0.05, it means no evidence on publication bias, otherwise, there would be a publication bias existing^39^. Statistical significance was 0.05 (P < 0.05) with 95% CI. Data were pooled and analysed by the software Comprehensive Meta-Analysis (version 2.0, Biostat, Englewood, New Jersey).

## Data Availability

All data generated or analysed during this study are included in this published article.
